# Upcoding in medicare: where does it matter most?

**DOI:** 10.1186/s13561-023-00465-4

**Published:** 2024-01-02

**Authors:** Keith A. Joiner, Jianjing Lin, Juan Pantano

**Affiliations:** 1 University of Arizona, Tucson, USA; 2 Russell Sage Lab 3203, Rensselaer Polytechnic Institute, 110 8Th St, Troy, NY 12180 USA

**Keywords:** Upcoding, Medicare improper payments, Medicare audits, Prospective payment system, Hospital admissions, I11, I13, H51

## Abstract

Upcoding in Medicare has been a topic of interest to economists and policy makers for nearly 40 years. While upcoding is generally understood as “billing for services at higher level of complexity than the service actually pro- vided or documented,” it has a wide range of definitions within the literature. This is largely because the financial incentives across programs and aspects under the coding control of billing specialists and providers are different, and have evolved substantially over time, as has the published literature. Arguably, the primary importance of analyzing upcoding in different parts of Medicare is to inform policy makers on the magnitude of the process and to suggest approaches to mitigate the level of upcoding. Financial estimates for upcoding in traditional Medicare (Medicare Parts A and B), are highly variable, in part reflecting differences in methodology for each of the services covered. To resolve this variability, we used summaries of audit data from the Comprehensive Error Rate Testing program for the period 2010–2019. This program uses the same methodology across all forms of service in Medicare Parts A and B, allowing direct comparisons of upcoding magnitude. On average, upcoding for hospitalization under Part A represents $656 million annually (or 0.53% of total Part A annual expenditures) during our sample period, while up- coding for physician services under Part B is $2.38 billion annually (or 2.43% of Part B annual expenditures). These numbers compare to the recent consistent estimates from multiple different entities putting upcoding in Medicare Part C at $10–15 billion annually (or approximately 2.8–4.2% of Part C annual expenditures). Upcoding for hospitalization under Medicare Part A is small, relative to overall upcoding expenditures.

## Introduction

Upcoding in Medicare has been a topic of interest to economists and policy makers for nearly 40 years. Upcoding is a term that is not defined in the regulations but is generally understood as “billing for services at a higher level of complexity than the services actually provided or documented.”^1^ In fact, its definition in the literature is largely applied by researchers with respect to the specific context of evaluation. Accordingly, the processes and framework for billing and the mechanisms to capture upcoding have changed markedly over the last four decades, as have the establishment and enrollment in different parts of Medicare. These include, but are not limited to, reforms of the Inpatient Prospective Payment System (IPPS), the advent of electronic medical records (EMRs), more rigorous audits of billing records, and the rapidly growing popularity and enrollment in Medicare Part C or Medicare Advantage (MA). Concomitantly the economics literature analyzing upcoding in Medicare has changed in focus, and emphasis. Arguably, the primary importance of analyzing upcoding in different parts of Medicare is to inform policy makers on the magnitude of the process and to suggest approaches to mitigate the level of upcoding. This is not a “one size fits all” undertaking, since the mechanisms for documenting the level of service through preparation of claims varies markedly when Medicare Parts A, B, C and D are compared. They are fundamentally different in terms of the metrics used and measured to determine the magnitude of the claim, a situation which is not typically highlighted in the litera- ture and not oftentimes appreciated. Another reason that could make it hard to provide a unified definition of upcoding across different types of services is due to the conflation of complexity and inefficiency in the delivery of healthcare services. One of the important categories of inefficiency in health care is additional spending on healthcare goods and services that lack evidence of improving health outcomes [15]. Thus, the complexity claimed could arise from inefficient delivery of medical services—such as overuse of diagnostics or overuse of therapeutics with no medical benefit—or inappropriately documented complexity, both being hard to distinguish from one another. Moreover, the extent to which this issue complicates upcoding varies across different types of Medicare services. Taken together, this is precisely why any general extrapolation on the relative importance of upcoding is not pos- sible without using some common metric. The one parameter that can be directly compared is the annual magnitude, in dollars, of upcoding, particularly when expressed as a percentage of total expenditures. While some estimates can be extrapolated from the reports from the Centers for Medicare and Medicaid Services (CMS), to our knowledge, no manuscript in the peer-reviewed literature has done so using data extending over decades, including recent time points. Thus, our paper contributes to the literature by using the data from the Comprehensive Error Rate Testing (CERT) program, which allows for a common approach to identify relative magnitude of upcoding in Medicare Parts A and B. The findings could provide potentially important implication—where attention is needed most to mitigate the level of upcoding.

Coding for Medicare Parts A and B is based on services provided to individual patients. Part A services include hospitalization, the largest component, and non-inpatient services, including skilled nursing facility care, and nursing home, home health and hospice care. Coding for each component of Part A services uses a different process, con- founding direct comparisons. Medicare Part B covers physician services, the largest component, and Part B durable medical equipment, prosthetics/orthotics, and supplies (DMEPOS). The parameters and processes for coding and gen- erating claims for each of these services are distinct, making comparisons about the magnitude of upcoding for each difficult at best [14].

The literature and focus on upcoding in Medicare has changed dramatically in the last several decades. Initial interest in upcoding, with papers as early as 1985, focused on upcoding for hospital admissions, following introduction of the IPPS in 1983, described in detail later in the paper. Upcoding as applied to hospitalization, was when a provider submitted bills/claims with diagnosis codes for more severe conditions than justified or documented [7, 21]. Analysis focused on hospital claims for Medicare Part A, in part because enrollment in Medicare Part C was a minor fraction of total Medicare enrollment. As late as 2015, the published literature on upcoding in Medicare was dominated by analyzing this form of overbilling.

Other forms of overpayment are sometimes conflated with upcoding because they are not predicated on incorrect or inadequate documentation [2]. These include billing for services not performed at all, unnecessary admissions, illegal referrals or kickbacks, and prescribing excessive tests or conducting excessive care. Billing for services not performed at all, and illegal referrals or kickbacks, are outright fraud. Unnecessary admissions are not a consequence of incorrect codes, but rather a decision on where the services are conducted. Prescribing excessive tests or conducting excessive care is typically used to either substantiate a diagnosis or rule out alternative diagnoses, and as such is neither incorrect or inadequate documentation, although in selected cases may be considered upcoding by CMS.^2^ Note that the overuse of medical services could result in overpayments, but it is primarily due to inefficiency, rather than complexity in medical conditions, reflecting the conflation of inefficiency and under-evidenced complexity we mentioned above. With implementation of the IPPS by Medicare in 1983, each admission is assigned a diagnosis-related group (DRG) code, based on a diagnosis or procedure. Coding for IPPS is typically based on the discharge summary. This is a description of the hospital admission, that includes a list of the primary and secondary diagnoses, procedures, a narrative account of the hospital course including the reason for admission, discharge medications, and ancillary data such as laboratory values, imaging results, notes from consulting services, and more. Typically, medical coders “select” a base DRG from the list of diagnoses/procedures in the discharge summary. A base DRG records the patient‘s primary diagnosis or procedure. Often, several DRGs share a common base DRG. For instance, DRGs 637 — 639 are “Diabetes with major complication or comorbidity (MCC),” “Diabetes with complication or comorbidity (CC),” and “Diabetes without CC/MCC,” respectively. All three belong to the same base DRG – diabetes. The base DRG can be modified depending on the presence or absence of a CC or MCC, which increases the weight of the DRG and hence the reimbursement. Justification for selecting a specific base DRG, with or without a CC or MCC, depends on appropriate and adequate documentation. The following are processes that constitute upcoding for IPPS and are the source for scrutiny by auditors: (1) selecting a base DRG with a higher weight than justified, (2) coding a CC or MCC modifying the base diagnosis that is not present/not sufficiently documented, (3) coding selected diagnoses as present on admission when they were not, and (4) unbundling services/procedures that are bundled under a single DRG. When any of these is done intentionally, it is fraud, and results in substantial financial penalties, sanctions, and even imprisonment. If unintentional, or inadver- tent, and if upheld on appeal, Medicare recoups the overpayment. While the consequence is that hospitals, providers and coders are highly attuned to the implications of improper coding, this does not preclude doing so if the incentives are sufficient. Importantly, upcoding for inpatient care under Medicare Part A is based on documentation of acute events precipi-tating admission, rather than on chronic conditions of the sort contributing to Hierarchical Condition Category (HCC) scores for MA plans, as discussed further below. This was the primary consequence and intent of the Medicare reform in 2007-2008. Prior to that reform, many common chronic conditions in an individual patient, such as diabetes (a con- tributor to the HCC score for MA plans), and in which details and documentation of acute problems were not specified, would increase DRG-based reimbursement. Following the reform, only documentation of some acute manifestation (such as gangrene in a foot ulcer in patients with diabetes) would lead to higher DRG-based reimbursements [20].

The magnitude of upcoding for inpatient admissions under Medicare Part A has only been estimated in a limited number of papers [6, 7, 9, 12]. Dafny [7] estimated that the annual payments increased by $330-425 million due to a policy of code change in 1988, or approximately 0.6% of the hospital PPS expenditures in 2000. Cook and Averett [6] concluded that an additional 3% in reimbursements could be associated with upcoding during the period following the 2007 reform, although the possibility that increased reimbursements reflected more accurate coding was not addressed. This is an important distinction, because the 2007 reform required increased effort on documentation to justify CCs and MCCs. In this regard, Gowrisankaran et al. [12] found negligible evidence of upcoding following the reform, but found that re- imbursements in Medicare Part A could increase by 0.8% given a unit change in DRG weights before the reform, which is in line with Dafny [7]. Importantly, Gowrisankaran et al. [12] concluded that more accurate coding rather than upcoding explained an increase of $1.08 billion following the reform, or 0.86% of the total hospital IPPS expenditures. Ganju et al. [9] considered the effects of auditing as a strategy to identify upcoding and suggested that the implementation of the recovery audit program saved approximately 0.80 % of (or $1 billion in) Medicare reimbursements. Each of these studies identifies upcoding using different methodologies to compare DRG weights for specific conditions, without any direct comparison for entire populations as possible with MA, as explained below.

Coding for skilled nursing facility care, nursing home care, home health care, and hospice care, covered under Part A non-IPPS, is not dependent on the diagnosis, but rather on the level of services provided and resources used. Reimbursement can be fee for service (FFS) or per diem, but in either case upcoding reflects inadequate/fraudulent documentation of the services delivered [3].^3^

Upcoding for physician services through Medicare Part B occurs when the provider submits claims for a more complex set of services provided than justified, independent of the diagnosis. The majority of these Part B claims are for ambulatory visits, where claims are prepared using evaluation and management (E&M) codes. Until recently, there were 5 levels of service for new patient visits, and an analogous set of 5 levels for established patient visits. Coding was dependent on the level of documentation of history, physical, laboratory data, and management plan, or on time spent in specific elements of the visit, using the assumption that provider effort (time) was proportionate to the level of documentation. Claims can now be coded solely based on the time for the visit, or on medical decision making, with only four levels for new or established patients [19]. Studies looking at upcoding for physician services have evaluated the distribution of levels service for similar populations of patients before and after changes in the reimbursement weights, at the frequency with which some providers use only the highest E&M codes, or at the plausibility of time-based codes [4, 8, 16]. In other words, just as for studies quantifying upcoding for Medicare Part A, the methodologies are substantially different, confounding direct comparisons. Moreover, it is even harder to identify whether the inflated complexity, if any in the bill, is due to the inefficiency in delivering healthcare services or inappropriate documentation, given that the reported complexity is mainly based on providers’ time and efforts.

Part B DMEPOS covers a wide range of items. Devices for improving respiratory function, equipment to assist with ambulation, infusion equipment and nerve stimulators for pain modulation. In order, the three most widely prescribed are continuous positive airway pressure devices, crutches, and humidifiers. Coding claims for these items is dependent on both the diagnosis and justification for need. Upcoding is most commonly based on inadequate or fraudulent documentation of need.

An increasing attention has been focused on upcoding for Medicare Part C, or Medicare Advantage [10, 11, 13], but the recognition of overpayments to MA plans is not new, going back more than 20 years [1]. Overpayments are estimated by comparing the expenditures for equivalent levels of service across large populations in MA and traditional Medicare Parts A and B, which cover the same services. Estimates have consistently been in the range of 10-14% greater reimbursements for also be challenging to separate under-evidenced complexity from the inefficiency in the provision of care. The method we use in this paper helps identify the relative magnitude of upcoding in different types of Medicare services but also highlights the challenge to combat upcoding in services other than Part A hospitalization care.

MA. In MA, insurance companies assume the financial risk for providing healthcare for a population of patients who enroll with them for Medicare coverage. The allocation from CMS to private insurers administering MA plans is based on the aggregate Hierarchical Condition Category (HCC) score for the population, derived from the accumulation of chronic conditions for all individuals covered by the plan. The HCC score is intended to capture the costs of all patient care over a year-long period [22]. This approach is needed to adequately risk adjust the payments, and preclude “cherry-picking” of healthier patients by the plans. The capitated payment to the MA plan, covering the population of enrolled payments, is then used to reimburse for individual patients’ services, including inpatient admissions and outpatient care.

Upcoding for MA reflects excessive or even fraudulent documentation of underlying chronic health conditions for any given individual. This can occur through a number of mechanisms, involving both providers and private insurers. When this occurs for a substantial number of individuals covered by any MA plan, the greater the capitated reimbursement from Medicare. Reviews of fraud lawsuits, inspector general audits, and watchdog investigations, detailed how the majority of large health insurers used this mechanism to expand profits. The estimated amount of payments associated with MA upcoding varies, depending upon the methodology, but is in the range of $9–12 billion on an annual basis over the last decade [1, 10, 17]. For instance, according to the report to the Congress by the Medicare Payment Advisory Commission, the higher coding intensity in MA plans could have resulted in at least $12 billion, and as much as $25 billion, in excess payments to MA plans in recennt years [18]. In the final rule on MA overpayments, published in the Federal Register on February 1, 2023, CMS estimated that in Fiscal Year (FY) 2021 alone, over $15 billion in Part C overpayments were made, representing 4.2% of total Part C payments.^4^ These estimates tend to be internally consistent because the process used in the calculation is based on comparisons of total spending across whole populations, rather than on the range or specifics of services provided to individual patients. This is in contrast to the situation with Medicare Parts A and B, where the methodology is variable when different papers are compared. That is our logic for using a common methodology when analyzing upcoding in traditional Medicare Parts A and B, and one that provides the most accurate account of the financial magnitude of upcoding.

## Comparing upcoding for Part A and Part B services – the Comprehensive Error Rate Testing Program

As explained above, the processes and mechanisms for upcoding the various services under Medicare Parts A and B vary widely. Upcoding can reflect incorrect/fraudulent diagnosis codes, provider effort independent of diagnosis, resources utilized, or justification of need. Estimates for the financial magnitude of each, let alone a comparison across services, are problematic. This is in the face of a predominant emphasis in the economic literature on upcoding in Part A.

We have utilized recently available reports summarizing the findings from the CERT program to circumvent this issue. Medicare uses and has used a variety of auditing strategies to evaluate the rate of improper payments. The only one that selects claims for auditing on a purely *random* basis is the CERT program. The other programs depend on algorithms that identify outliers to trigger a review, such as a substantial increase in the number of submitted claims or for a specific DRG from a single organization, or a red flag from the CERT program. According to CMS, an improper payment is defined as “any payment that should not have been made or that was made in an incorrect amount (including overpayments and underpayments) under statutory, contractual, administrative, or other legally applicable requirements” [5]. The fact that CERT audits are performed on a random sample of Medicare claims is essential to the results we present and to our conclusions. Using results from other audits would not represent the overall prevalence of improper bills, since they constitute a pre-selected subset flagged to trigger reviews. The CERT program selects at random approximately 50,000 claims from those submitted to Medicare during a reporting period. The CERT review contractor sends a request to the provider requesting that medical documentation be submitted for CERT review. CERT auditors review them and identify categories of improper payments. They neither have responsibility or authority to levy fines, nor do they negotiate with hospitals regarding the results of their reviews. The information gathered by a CERT contractor is also used to improve system edits, update coverage policies and manuals and conduct provider education efforts.

Claims are analyzed separately for Medicare Part A IPPS, Part A non-IPPS, Medicare Part B, and Part B DMEPOS. Claims are categorized as either proper payments or into one of five improper payment types: Medical necessity, no documentation, insufficient documentation, incorrect coding or other, using the stratification below. Of central importance, CMS emphasizes that improper billing falling under any of these categories should not necessarily be considered fraud.

- Medical necessity — The DRG diagnosis/procedure is for a service that should be carried out in an ambulatory setting rather than as inpatient.
- No documentation — The provider either fails on repeated requests to provide the medical records, or indicates they do not have the documentation.
- Insufficient documentation — Medical documentation is not adequate to justify that the services were provided.
  Specific required documentation elements that are missing, such as a physician signature, also place the claims in this category.
  Incorrect Coding — Documentation provided indicates that the wrong code was entered, the service was unbun- dled, or the wrong billing provider or site was listed.
- Other — A wide array of miscellaneous causes for improper bills.

### Improper payment errors in Part A IPPS

We first present the improper payment rates for Part A IPPS in 2010-2019 for different error types in Table 1 using summaries of results reported by the CERT program. As mentioned above, the category of improper payments cover any payment that was incorrect for any reason. They are not tantamount to fraud, and capture errors in across multiple categories, many of which are simple errors of omission. In the large majority of cases, improper bills regardless of the category are resolved by the claims adjudication, limiting the magnitude of any financial losses to the Medicare program.

**Table 1:** Error type distribution (%) in Part A IPPS

Fiscal Year	Incorrect coding	Insufficient documenta- tion	Medical necessity	No documen- tation	Other	Total
2010	1.04	1.14	7.28	0.12	0.03	9.60
2011	1.14	1.14	6.86	0.11	0.04	9.30
2012	1.87	0.90	7.03	0.00	0.09	9.89
2013	1.94	0.71	9.39	0.02	0.13	12.20
2014	1.16	1.09	5.08	0.00	0.07	7.40
2015	0.90	0.43	2.97	0.03	0.18	4.50
2016	1.43	0.44	2.45	0.01	0.07	4.40
2017	0.76	1.08	2.78	0.08	0.11	4.80
2018	0.78	0.88	2.48	0.04	0.02	4.20
2019	0.55	1.04	2.28	0.02	0.11	4.00

SOURCE: The reported improper payment rates in Part A IPPS in Medicare FFS Improper Payments Reports 2011 – 2020 [5].

The improper payment rate for a particular category is equal to the proportion of improper payments for that category to the total payments, with some weighting applied. Note that the sample is projected to the universe statistically using a combination of the sampling weight and the relative share of universe expenditures (Supplementary Appendices, [5]).

In all the years, the largest category is medical necessity (range 2.28% to 9.39%), followed by incorrect coding (range 0.55% to 1.94%) and insufficient documentation (range 0.43% to 1.14%).

Given the above, Table 1 suggests that the largest contributor to improper bills through IPPS arises from medical necessity, a type of improper billing that is more about where the medical service should be performed than incom- plete or fraudulent documentation of the diagnosis or service level provided. In fact, there is a dramatic decrease in medical necessity as a cause of improper claims, between 2014 and 2015. The most likely explanation relates to the implementation of the “two-midnight rule.” CMS has recognized an increasing number of patients being observed for extended stays in an outpatient setting (primarily observational units linked to emergency departments), with variabil- ity around the justification for an inpatient admission under IPPS. Therefore, CMS adopted the “two-midnight rule” in FY 2014, clarifying when inpatient hospital admissions are generally appropriate for Medicare Part A payments. According to this rule, inpatient admissions will generally be payable under Part A if the admitting practitioner ex- pects the patient to require a hospital stay that crosses two midnights and the medical record supports that reasonable expectation. Clarification was accompanied with a 72% decline in the fraction of IPPS claims considered improper due to medical necessity [5]. This sequence is highly illustrative: Improper bills seem to reflect changes in medical practice coupled with the complexity of coding, and not fraud.

### Improper payment errors in Part B Physician services

Total improper payment rates for Part B physician services average 10.5% over the period from 2010-2019 (Table 2). The most common reason for improper billing for Part B physician services is insufficient documentation. As explained above, coding is based on estimates of provider effort, independent of diagnosis.

**Table 2:** Error type distribution (%) in Part B

Fiscal Year	Incorrect coding	Insufficient documenta- tion	Medical necessity	No documen- tation	Other	Total
2010	2.95	6.52	0.36	0.65	0.01	10.49
2011	2.97	6.11	0.31	0.48	0.03	9.89
2012	2.70	6.88	0.38	0.45	0.09	10.50
2013	3.16	8.24	0.27	0.21	0.23	12.10
2014	3.12	8.66	0.22	0.48	0.22	12.70
2015	2.96	7.98	0.26	0.39	0.12	11.70
2016	2.76	6.69	0.35	0.31	0.09	10.20
2017	2.53	6.98	0.33	0.36	0.50	10.70
2018	2.58	5.27	0.27	0.37	0.11	8.60
2019	1.75	5.52	0.15	0.49	0.19	8.11

SOURCE: The reported improper payment rates in Part B IPPS in Medicare FFS Improper Payments Reports 2011 – 2020 [5].

We also provide the overall improper payment rates for other services in Medicare Parts A and B in Appendix in Table 3.

### Financial consequence of upcoding

Up to this point, we have explained the process by which upcoding could occur with claims submitted to CMS for hospitalization and physician services, and have shown the breakdown by category of improper claims. This informa- tion does not provide a direct assessment of the magnitude of upcoding. For example, upcoding for IPPS would most commonly reflect preferential selection of DRG codes with higher weights. This can result either from (incorrect) selection of base DRGs with higher weights than the correct base DRG, or from coding higher pay DRGs (those with CCs or MCCs), when CCs or MCCs are not present, or from unbundling services that are included under a single DRG. These distinctions cannot be made from improper payment rates, since as stated earlier, improper payments are not necessarily tantamount to fraud.

**Fig. 1:**
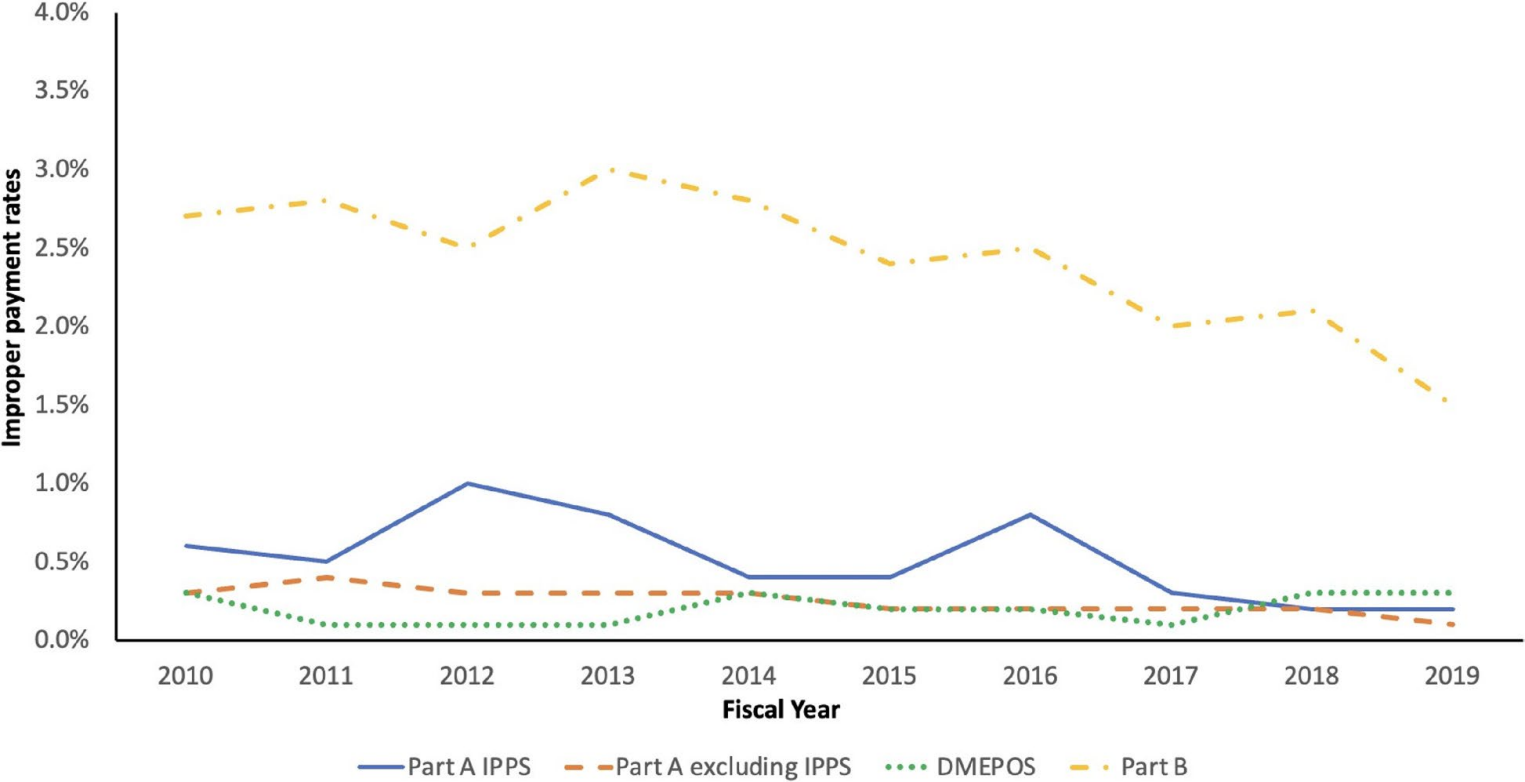
Improper payment rates due to upcoding by claim types

To determine the magnitude of upcoding, we use the data summarized in the CERT report provided by CMS on upcoding specifically for IPPS and other components of Medicare Part A and B. Figure 1 depicts the reported improper payment rates due to upcoding during the sample period, separately for each claim type. Upcoding constitutes 1% or less (average of 0.53%) of total reimbursements for IPPS over the period from 2010-2019. On average, the projected improper payment associated with IPPS upcoding is $656 million per year.^5^ Of particular importance, other processes account for 75-95% of improper bills for IPPS (Compare IPPS in Figure 1 and the last column of Table 1). Specifically, the large majority of the financial costs of improper billing are for medically unnecessary admissions (Table 1).

According to the summary reports from the CERT program, the average projected amount associated with upcod- ing for physician services under Part B is $2.38 billion per year (or 2.43% of Part B annual expenditures). Upcoding percentages for Part A non-IPPS and Part B DMEPOS is less than 0.27% on average, and together accounts for only $447 million annually.

## Discussion and conclusion

Our results allow direct comparison of improper payment rates and upcoding for all components of Medicare Parts A and B. These can be expressed as a percentage of claims between the various services, and by the financial impact. The estimated combined financial impact of upcoding, on average over the period 2010-2019, is approximately $3.48 billion per year, predominantly due to upcoding in Medicare Part B. This value is substantially lower than the estimates of $9-12 billion for years for MA. Although differences in methodology preclude direct comparisons, it is nonetheless likely that upcoding for Parts A and B has a smaller financial impact than for MA.

According to CERT reports, the annual projected amount from Part A IPPS upcoding is $656 million (0.17% of total Medicare Parts A and B expenditures).^6^ In combination, these data suggest upcoding of hospital admissions is minor in frequency and in financial impact compared to the totality of other concerns. We believe that more stringent regulatory initiatives starting in 1996, reforms of the DRG system, and widespread adoption of EMRs, could contribute to the low incidence of upcoding in Part A hospital inpatient care.

Note that it is likely that the CERT program does not detect all upcoding in practice, but neither does it detect all other forms of improper billing. The focus on our paper is not the precise incidence, prevalence or financial impact of upcoding for hospital admissions or other services covered by Medicare Parts A and B, but rather the *relative* contribution of each to the overall universe of improper billing, and upcoding. There is no reason to assume that there is preferential attention to one explanation for improper billing by CERT auditors, nor that upcoding is more difficult to identify than other forms of improper billing. In fact, medical necessity is the category with the most ambiguity, given the complexity of the clinical situation leading to the decision to admit a patient to the hospital.

CMS advises very directly that the CERT data cannot be used as an indication of fraud. The data does not distin- guish between intentional and unintentional upcoding, the latter of which may result in payment adjustments, but not in prosecutions. It is out of the scope of this study to respectively characterize “incidental” upcoding and “intentional” upcoding—both of them lead to additional financial expenses. Nor is it within the scope of this paper to differentiate between other forms of incidental vs intentional improper billing, all of which lead to additional financial expenses. In the large majority of cases, improper bills regardless of the category are resolved by the claims adjudication, limiting the magnitude of any financial losses to the Medicare program.

There are a few caveats to our study. First of all, we base our primary analysis on the CERT data. The results could be biased if the sample is poorly constructed or the incentives of auditors are misaligned. We do not view both cases as substantial concerns because CMS has been using the current strategy for sample selection since 2012 (the time period of our study). While we do not obtain much information on the payment schemes for CERT auditors, we believe that they tend to review claims rigorously as they are independent auditors that neither have responsibility nor rights to levy fines from hospitals, nor do they interact directly with hospital personnel. Moreover, CMS uses the result from CERT in a wide range of settings to improve the reimbursement schemes and policies, not simply to identify improper bills. Finally, it is likely that the CERT auditors may not capture every instance of upcoding. However, our paper focuses on the relative contribution of upcoding from different components in Medicare Parts A and B services to the overall universe of improper billing, which we show is quite low for Medicare Part A IPPS. Based on our results, the current regulatory processes to minimize upcoding for hospital admissions under Part A IPPS are highly effective. To further mitigate the problem of upcoding in other parts of Medicare services, it would be important to first address the challenge of the conflation between under-evidenced complexity and inefficiency in healthcare delivery.

## Data Availability

The datasets generated and/or analysed during the current study are available in this link: https://www.cms.gov/research-statistics-data-and-systems /monitoring-programs/medicare-ffs-compliance-programs/cert/cert-reports.

## Appendix

**Table 3:** Total improper payment rate (%) in other Parts A and B services

Fiscal Year	Part A non-IPPS	Part B DMEPOS
2011	4.80	66.0
2012	8.20	58.2
2013	13.1	53.1
2014	14.7	39.9
2015	14.0	46.3
2016	11.3	44.6
2017	8.10	35.5
2018	8.10	30.7
2019	6.20	31.8

SOURCE: Data directly obtained from the reported improper payment rates for these services in Medicare FFS Improper Payments Reports 2011 – 2020 [5].

## Abbreviations

IPPS: Inpatient Prospective Payment System
EMR: Electronic medical record
MA: Medicare Advantage
CMS: Centers for Medicare & Medicaid Services
DMEPOS: Durable medical equipment, prosthetics/orthotics, and supplies
DRG: Diagnosis related group
MCC: Major complication or comorbidity
CC: Complication or comorbidity
HCC: Hierarchical Condition Category
FFS: Fee for service
E&M: Evaluation and management
FY: Fiscal Year
CERT: Comprehensive Error Rate Testing

## Footnotes

1 See https://www.cms.gov/files/document/overviewfwacommonfraudtypesfactsheet072616pdf.

2 For instance, see https://www.ama-assn.org/system/files/issue-brief-cms-modifier-25.pdf.6pdf.

3 Note that complexity of care under Part A non-IPPS is defined in a similar way to that in Part B, or Part C non-hospitalizations. Thus, it could.

4 Note that the FY is the accounting period for the federal government, from the fourth quarter of the previous year to the third quarter of the current year.

5 We deflate the dollar amounts in different years using the corresponding Medicare Economic Index to make sure the amounts across different years are comparable. We do this for all the average payment amounts discussed in this paper. We obtain the Medicare Economic Index from CMS (https://www.cms.gov/research-statistics-data-and-systems/statistics-trends-and-reports/medicareprogramratess tats/marketbasketdata).

6 On average, the total expenditures on Medicare Parts A and B are $388 billion per year between 2010 and 2019.
